# Assessment of a Transverse-Sigmoid Sinus Dural Arteriovenous Fistula using Transcranial Doppler Ultrasonography: A Case Report

**DOI:** 10.2174/0115734056473694260723114721

**Published:** 2026-08-05

**Authors:** Xiangxiang Peng, Shan Geng, Zhang Xiong, Zhiming Zhou

**Affiliations:** 1 Department of Neurology, The First Affiliated Hospital of Wannan Medical College (Yijishan Hospital), Wuhu, Anhui Province, China;; 2 Department of Neurology, Beijing Tiantan Hospital, Capital Medical University, Beijing, China;; 3 Department of Neurosurgery, The First Affiliated Hospital of Wannan Medical College (Yijishan Hospital), Wuhu, Anhui Province, China

**Keywords:** Arteriovenous fistula, Transverse-sigmoid sinus, Transcranial doppler, Ultrasound, Digital subtraction angiography, CT

## Abstract

**Background:**

The most common symptoms of transverse-sigmoid sinus dural arteriovenous fistula (TS-S DAVF) are pulsatile tinnitus and headache. The clinical characteristics lack specificity and may lead to misdiagnosis or delayed diagnosis. The noninvasive and convenient nature of neurovascular ultrasound has made it an important screening tool for lesions. This paper reports the use of transcranial Doppler (TCD) ultrasonography for the preoperative screening and postoperative follow-up of a patient diagnosed with a transverse-sigmoid sinus dural arteriovenous fistula (TS-S DAVF).

**Case Presentation:**

A 62-year-old female was admitted with a six-month history of headache and tinnitus on the left side. A structured TCD-based approach was used to evaluate intracranial and extracranial hemodynamics. The protocol included targeted probe positioning based on anatomical landmarks, spectral waveform analysis, and the superficial temporal artery tap maneuver. The preoperative TCD results indicated hemodynamic alterations with increased flow and decreased resistance in the left common carotid artery, external carotid artery, and occipital artery. The presence of a Cognard Type III DAVF in the left transverse sinus-sigmoid sinus was confirmed through DSA. The occipital artery and the posterior branch of the middle meningeal artery were the key feeding arteries with cortical vein drainage to the sigmoid sinus. After endovascular embolization *via* the venous approach, follow-up TCD showed normal hemodynamics in the previously affected arteries, consistent with complete resolution of the patient’s clinical symptoms.

**Conclusion:**

A structured TCD-based approach may help identify shunt-related hemodynamic abnormalities in suspected TS-S DAVF and support post-embolization follow-up. Cerebral DSA remains necessary for definitive diagnosis and treatment planning.

## 1 INTRODUCTION

Intracranial dural arteriovenous fistula (DAVF) is a rare cerebral vascular malformation. Its spontaneous resolution rate is extremely low [1-3]. Without timely intervention, DAVF may lead to severe neurological deficits or fatal outcomes [1, 2, 4]. The transverse-sigmoid sinus is one of the most commonly involved sites [5], and transverse-sigmoid sinus dural arteriovenous fistula (TS-S DAVF) often presents with pulsatile tinnitus and headache [6]. Because these symptoms are nonspecific, diagnosis may be delayed or missed [7].

Digital subtraction angiography (DSA) is the gold standard for diagnosing DAVF and characterizing its angioarchitecture. However, its invasive nature and resource requirements make it less suitable for routine initial screening. Non-contrast head CT may show secondary changes, whereas CT angiography and magnetic resonance imaging can provide useful vascular information but may be limited by invasiveness, cost, and accessibility [8, 9].

Neurovascular ultrasound can provide rapid, non-invasive hemodynamic information. Its potential value lies in detecting an indirect hemodynamic pattern known as “pseudo-internalization”. The pattern is characterized by increased end-diastolic velocity (EDV) and reduced resistance index [10]. Although transcranial Doppler (TCD) ultrasonography is widely used for cerebrovascular hemodynamic assessment, its role in DAVF evaluation remains limited because the fistulous connection and venous angioarchitecture cannot be directly visualized. We report a rare case of transverse-sigmoid sinus dural arteriovenous fistula (TS-S DAVF) in which a structured TCD-based approach contributed to initial hemodynamic screening and postoperative follow-up.

## 2 CASE PRESENTATION

### 2.1 Clinical Presentation

A previously healthy 62-year-old female presented with a 6-month history of left-sided headache and pulsatile tinnitus. On physical examination, a continuous thrill was palpable over the left mastoid region. Auscultation with a bell-shaped stethoscope revealed a blowing vascular bruit that continued through systole and diastole.

### 2.2 TCD Examination Protocol

TCD assessment was performed using a multi-frequency system (Doppler-Box, DWL, Germany), with a 2-MHz pulsed-wave transducer for intracranial evaluation and a 4-MHz continuous-wave transducer for extracranial hemodynamic assessment. The patient was examined in the supine position, with the examiner seated behind the patient’s head and the monitor positioned toward the examiner.

Extracranial vascular assessment focused on the common carotid, internal carotid, external carotid, and occipital arteries. Because continuous-wave Doppler does not provide range-gated depth resolution, no fixed sample depth or sample volume was used. The probe was positioned along the expected anatomical course of each target vessel and adjusted until a stable Doppler spectrum was obtained. The wall filter was generally set at 50-100 Hz, and gain was optimized to produce a complete spectral envelope, a clear spectral window, and minimal background noise. Vessels were identified by anatomical location, flow direction, waveform morphology, and response to dynamic maneuvers. The superficial temporal artery tap maneuver, performed just anterior to the tragus and above the zygomatic arch, helped distinguish the external carotid artery from the internal carotid artery; rhythmic sawtooth-like oscillations in the Doppler spectrum further supported external carotid artery identification. The occipital artery was examined along its superficial course posterior to the auricle and around the mastoid region. M-mode imaging, when available, was used as an adjunct for vessel localization.

Intracranial arterial assessment was performed with the 2-MHz pulsed-wave transducer through standard acoustic windows according to established sonographic protocols [11].

### 2.3 Surgical Procedure

DSA was performed *via* percutaneous femoral artery puncture under local anesthesia and confirmed a left TS-S DAVF. The main feeders were the OA and the posterior branch of the middle meningeal artery, with direct cortical venous drainage without venous ectasia, consistent with a Cognard type III lesion [12]. After shared decision-making with the patient, endovascular embolization was performed under general anesthesia *via* a transvenous approach through the internal jugular vein, sigmoid sinus, and transverse sinus [13]. Under fluoroscopic guidance, 2.3 mL of Onyx-18 was injected. Control angiography confirmed complete occlusion of the fistula without residual shunting, as shown in Fig. (**1**).

### 2.4 Preoperative TCD Findings

Vessel identification was confirmed using the structured TCD protocol described above. Hemodynamic comparison showed elevated EDV and reduced PI in the left CCA, together with markedly increased PSV and EDV and reduced PI in the left ECA and OA. These findings indicated a high-flow, low-resistance pattern in the left extracranial arterial system, most prominently involving the ECA and OA. Although the temporal window was suboptimal, combined temporal and transorbital insonation did not reveal any definite intracranial vascular abnormality in the examined segments. Overall, the findings suggested abnormal arteriovenous shunting and raised suspicion for TS-S DAVF. Representative preoperative Doppler spectra are shown in Fig. (**2**).

### 2.5 Postoperative TCD Findings

Follow-up TCD was performed 7 days after embolization. Compared with the preoperative examination, the EDV of the left CCA and the PSV and EDV of the left ECA and OA decreased markedly, whereas the PI values of these vessels increased and became more comparable to those of the corresponding contralateral arteries. The detailed preoperative and postoperative TCD-derived hemodynamic parameters are summarized in Table **1**. Representative postoperative Doppler spectra are shown in Fig. (**3**). Figure **4** illustrates the corresponding pre- and postoperative changes in EDV, PI, PSV, and mean flow velocity (MFV). These findings are consistent with attenuation of shunt-related hemodynamic disturbance after embolization.

## 3 DISCUSSION

This case demonstrates a high-flow, low-resistance Doppler pattern in the extracranial feeding arteries in TS-S DAVF, with partial reversal of these abnormalities after embolization. Reports focusing on a structured TCD-based approach for both screening and post-treatment follow-up of TS-S DAVF remain limited. Previous ultrasound-based studies have largely relied on carotid duplex sonography, transcranial color-coded Doppler sonography, or combined neurovascular ultrasound approaches [10, 14-17]. Because TCD cannot directly visualize the fistulous connection or venous angioarchitecture, these findings should be regarded as indirect hemodynamic evidence rather than definitive diagnostic proof. Even so, a structured TCD-based examination may serve as a practical adjunct for identifying shunt-related hemodynamic abnormalities and monitoring post-embolization flow changes.

In this patient, preoperative TCD showed a high-flow, low-resistance pattern in the left CCA, ECA, and OA, mainly reflected by elevated EDV and reduced PI. The changes were most marked in the ECA and OA, suggesting increased extracranial arterial inflow related to the fistula. On follow-up TCD performed 7 days after embolization, flow velocities decreased and PI increased in the affected vessels, indicating partial reversal of the preoperative shunt-related hemodynamic pattern. This pattern is in line with previous Doppler-based studies showing that arteriovenous shunting lesions may produce high-velocity, low-resistance waveforms in feeding arteries [14, 18]. Hemodynamic changes in the OA may be particularly informative when this artery contributes to the supply of a lateral sinus DAVF [14]. A recent report of ethmoidal DAVF further showed that Doppler changes in a feeding artery may parallel angiographic findings during treatment and follow-up [19]. These findings support the potential value of Doppler-based hemodynamic monitoring after embolization.

An important aspect of this case was that the TCD findings were interpreted within a structured examination framework rather than on the basis of a single Doppler parameter. Extracranial vessel identification relied on anatomical landmarks, flow direction, waveform morphology, and dynamic verification maneuvers. The superficial temporal artery tap maneuver was particularly useful for distinguishing ECA-derived flow from ICA flow. Rhythmic tapping over the superficial temporal artery produced sawtooth-like oscillations in the ECA and ipsilateral OA waveforms, whereas the ICA showed little or no response. Together with the low-resistance, high-flow ECA waveform resembling “pseudo-internalization,” these findings provided supportive hemodynamic evidence of abnormal arteriovenous shunting [10, 18, 20].

Beyond vessel identification, this case underscores the role of hemodynamic assessment in the evaluation of arteriovenous shunting disorders. However, the extracranial Doppler abnormalities identified in this patient are not specific to TS-S DAVF. Similar low-resistance Doppler waveforms in extracranial arteries may also be encountered in other high-flow vascular conditions, including hypervascular head and neck tumors, scalp arteriovenous malformations or fistulas, carotid-cavernous fistulas, and other extracranial arteriovenous shunting lesions [21-24]. Recognition of these overlapping Doppler signatures is essential to avoid misinterpretation of isolated ultrasound findings. Accordingly, DSA remains indispensable when DAVF is suspected or when detailed assessment of venous drainage architecture is required.

This report has several limitations. As a single case, it cannot determine the diagnostic accuracy or generalizability of this TCD-based approach. TCD provides indirect, operator-dependent hemodynamic information, and a normal examination cannot exclude DAVF, particularly in low-flow lesions with subtle or absent Doppler abnormalities. In addition, postoperative TCD was performed only once, 7 days after embolization, so immediate postprocedural changes and serial hemodynamic evolution could not be assessed. Larger studies using standardized protocols are needed to better define the role of TCD in the screening and follow-up of suspected TS-S DAVF.

## CONCLUSION

A structured TCD-based approach may provide useful non-invasive hemodynamic information in patients with suspected TS-S DAVF and may help monitor post-embolization flow changes. However, TCD should be regarded as an adjunctive screening and follow-up tool rather than a definitive diagnostic modality. Cerebral DSA remains essential for definitive diagnosis, angioarchitectural characterization, and treatment planning.

## Figures and Tables

**Fig. (1):**
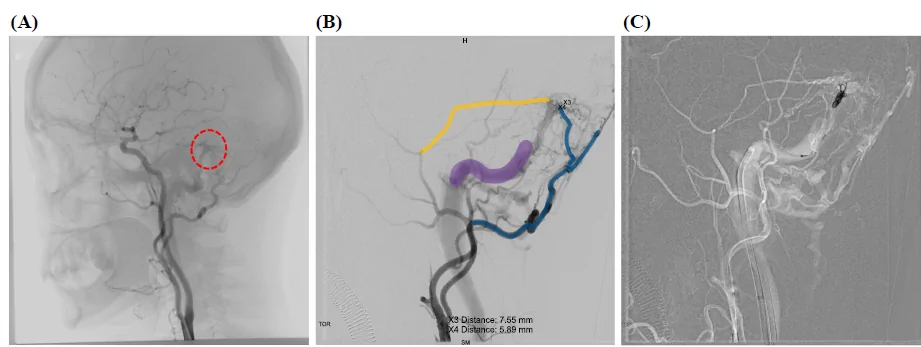
Cerebral angiography and embolization of a transverse-sigmoid sinus dural arteriovenous fistula. **A**. Transfemoral cerebral angiography demonstrating a dural arteriovenous fistula (red circle). **B**. Left external carotid artery cerebral angiography showing the dilated occipital artery (blue), middle meningeal artery (yellow), and early venous drainage into the sigmoid sinus (purple). **C**. Endovascular embolization *via* the internal jugular vein-sigmoid sinus-transverse sinus route using Onyx-18.

**Fig. (2):**
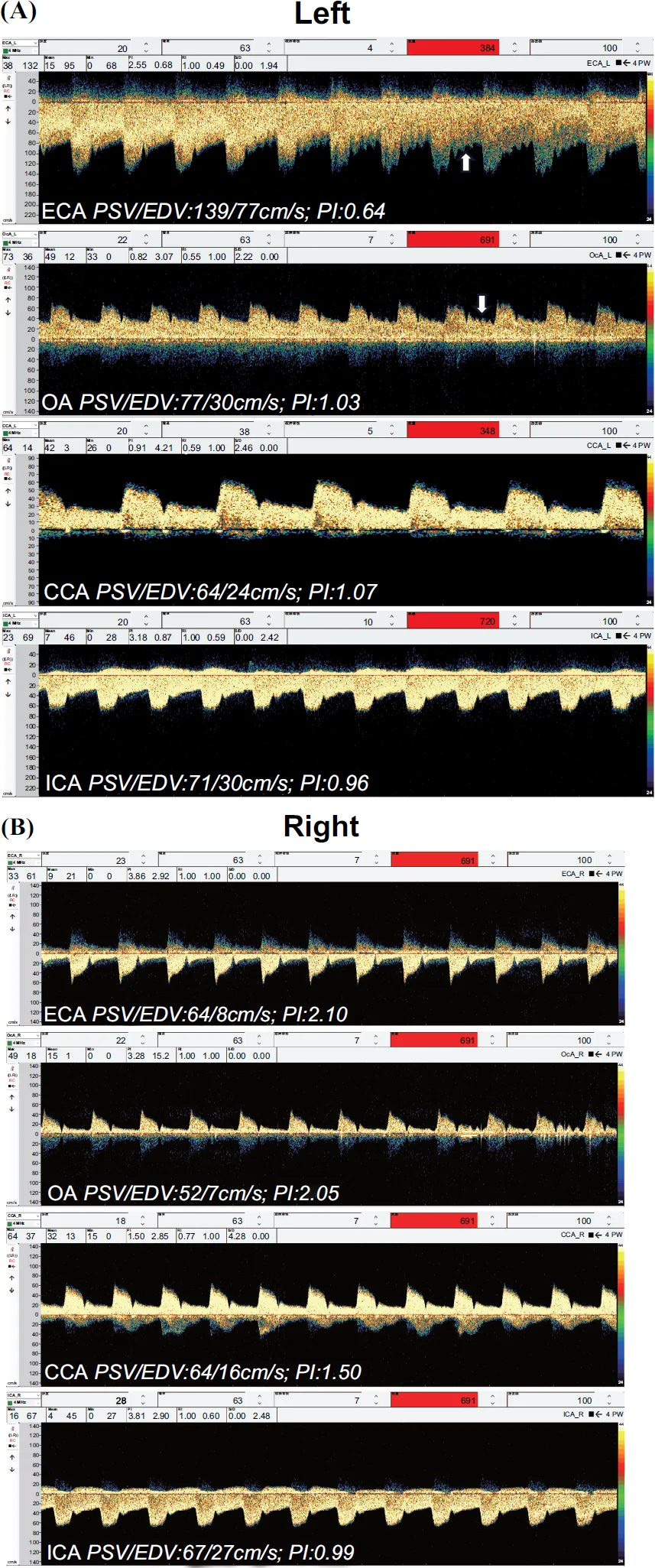
Preoperative TCD spectral waveforms and hemodynamic parameters of extracranial cerebral arteries. **A**. Left side: The left external carotid artery (ECA) and occipital artery (OA) exhibit a high-flow, low-resistance pattern with elevated end-diastolic velocity and reduced pulsatility index (PI). The characteristic “sawtooth” waves (white arrows) appear in the left ECA and OA spectra following the temporal artery tap maneuver. **B**. Right side: The right ECA and OA show normal flow velocities and PI compared with the left side. Values represent peak systolic velocity/end-diastolic velocity (cm/s) and PI. ECA, external carotid artery; OA, occipital artery; CCA, common carotid artery; ICA, internal carotid artery.

**Fig. (3):**
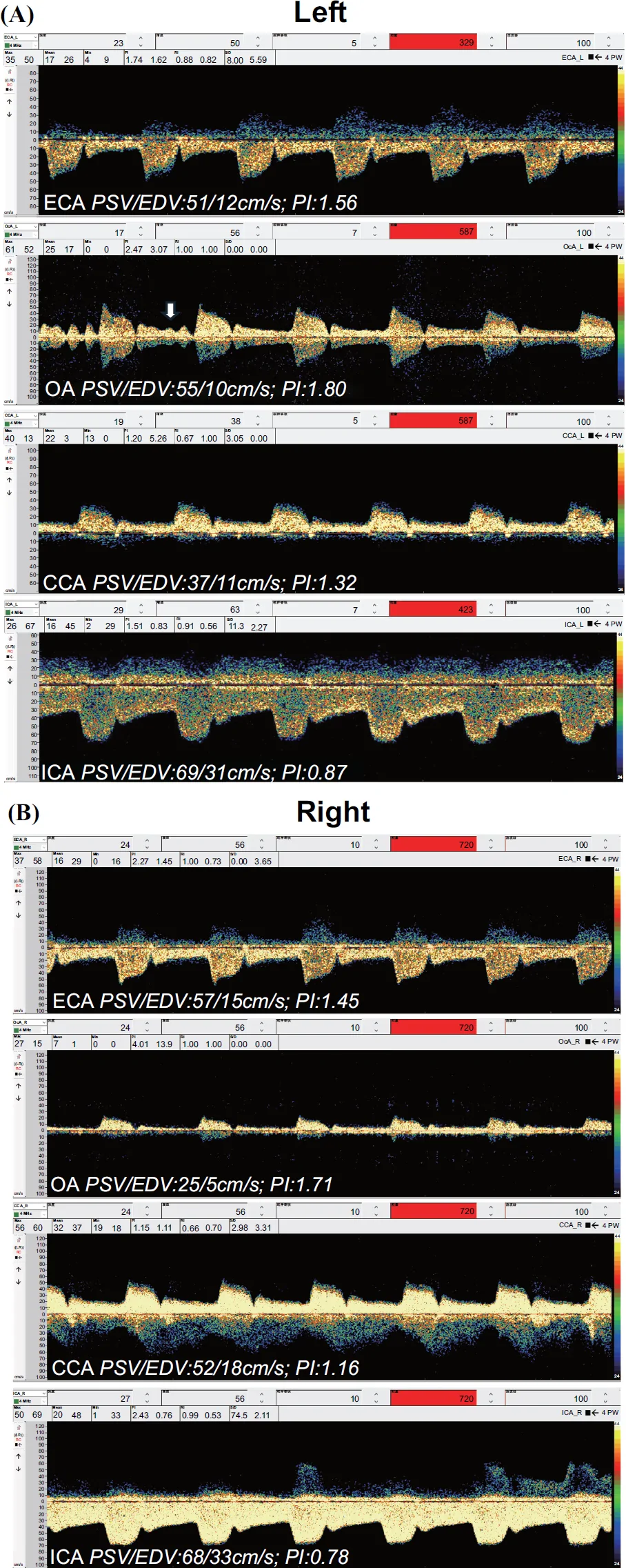
Postoperative TCD spectral waveforms and hemodynamic parameters of extracranial cerebral arteries. **A**. Left side: The pathological high-flow, low-resistance pattern in the left external carotid (ECA) and occipital (OA) arteries has resolved, with pulsatility indices now essentially symmetric with those on the contralateral side. All waveforms appear physiologically normal. The characteristic “sawtooth” waves (white arrows) appear in the left OA spectra following the temporal artery tap maneuver. **B**. Values represent peak systolic velocity/end-diastolic velocity (cm/s) and PI. ECA, external carotid artery; OA, occipital artery; CCA, common carotid artery; ICA, internal carotid artery.

**Fig. (4):**
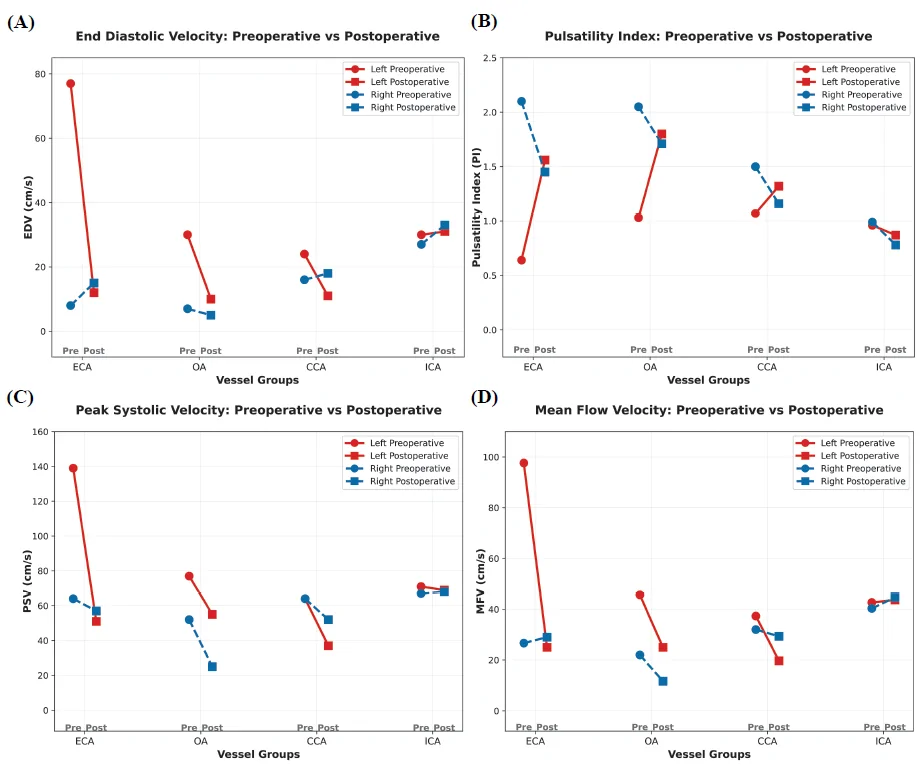
Preoperative and postoperative changes in extracranial TCD-derived hemodynamic parameters. Line graphs show changes in (**A**) end-diastolic velocity (EDV), (**B**) pulsatility index (PI), (**C**) peak systolic velocity (PSV), and (**D**) mean flow velocity (MFV) before embolization and 7 days after embolization. The left external carotid artery (ECA) and occipital artery (OA) showed reduced flow velocities and increased PI after embolization, consistent with attenuation of the preoperative high-flow, low-resistance pattern.

**Table 1:** Preoperative and postoperative Doppler-derived hemodynamic parameters of extracranial arteries.

**Vessel**	**Side**	**Preoperative PSV/EDV (cm/s)**	**Preoperative PI**	**Postoperative PSV/EDV (cm/s)**	**Postoperative PI**
ECA	Left	139/77	0.64	51/12	1.56
ECA	Right	64/8	2.10	57/15	1.45
OA	Left	77/30	1.03	55/10	1.80
OA	Right	52/7	2.05	25/5	1.71
CCA	Left	64/24	1.07	37/11	1.32
CCA	Right	64/16	1.50	52/18	1.16
ICA	Left	71/30	0.96	69/31	0.87
ICA	Right	67/27	0.99	68/33	0.78

**Abbreviations:** PSV, peak systolic velocity; EDV, end-diastolic velocity; PI, pulsatility index; ECA, external carotid artery; OA, occipital artery; CCA, common carotid artery; ICA, internal carotid artery. **Note:** Follow-up Doppler examination was performed seven days after embolization. Vessels are listed in the same order as in Figs. (**2** and **3**).

## Data Availability

The data and supporting information are included in the article.

## LIST OF ABBREVIATIONS

DAVF: Dural Arteriovenous Fistula
TS-S: Transverse-sigmoid sinus
TCD: Transcranial Doppler
DSA: Digital Subtraction Angiography
CT: Computed tomography
EDV: End-Diastolic Velocity
RI: Resistance Index
CCA: Common Carotid Artery
ICA: Internal Carotid Artery
ECA: External Carotid Artery
OA: Occipital Artery
PI: Pulsatility Index
PSV: Peak Systolic Velocity
MFV: Mean Flow Velocity

## STANDARDS OF REPORTING

CARE guidelines were followed.
